# Occurrence of gastrointestinal nematodes in lambs in Norway, as assessed by copromicroscopy and droplet digital polymerase chain reaction

**DOI:** 10.1186/s13028-024-00743-z

**Published:** 2024-05-25

**Authors:** Maiken Gravdal, Ian David Woolsey, Lucy Jane Robertson, Johan Höglund, Christophe Chartier, Snorre Stuen

**Affiliations:** 1 Department of Production Animal Clinical Sciences, Faculty of Veterinary Medicine, Norwegian University of Life Sciences, Svebastadveien 112, 4325 Sandnes, Norway; 2 Department of Paraclinical Sciences, Faculty of Veterinary Medicine, Norwegian University of Life Sciences, Elizabeth Stephansens Vei 15, 1433 Ås, Norway; 3 Department of Biomedical Sciences and Veterinary Public Health, Swedish University of Agricultural Sciences, Box 7028, 75007 Uppsala, Sweden; 4 BIOEPAR, INRAE, Oniris, 44307 Nantes, France

**Keywords:** ddPCR, Europe, *Haemonchus*, *Nematodirus*, Norway, Sheep, *Teladorsagia*, *Trichostrongylus*

## Abstract

**Background:**

Gastrointestinal nematodes (GINs) have a major impact on sheep production, health, and welfare worldwide. Norway is no exception, but there are only a few studies on the prevalence of GINs in Norwegian sheep. The aim of this study was to investigate the current occurrence of the most important nematodes in sheep flocks in Norway.

Faecal samples were collected from flocks in 2021/2022, mainly from three geographical regions in Norway, i.e., northern, eastern, and western. In each of 134 flocks included, individual samples from 10 lambs (autumn) were pooled. Third stage larvae (L3) were cultivated and harvested (Baermann method) from the pooled samples. The DNA was then extracted and further analysed using droplet digital PCR (ddPCR). This enables assessment of the proportions of the three most important nematode species/genera, i.e., *H. contortus*, *T. circumcincta*, and *Trichostrongylus*. The fractional abundance/relative proportion of each species/genus was assessed by performing duplex assays with universal strongyle and species/genus-specific primers and probe sets. In addition, the occurrence of *Nematodirus* eggs was assessed by standard faecal egg counts (i.e., McMaster method).

**Results:**

Of the 134 flocks sampled, 24 were from the northern region, 31 from eastern, and 71 from western Norway. In addition, some flocks from central (n = 7), and southern (n = 1) Norway were included. Among the sampled flocks, *T. circumcincta* occurred most commonly (94%), followed by *H. contortus* (60%) and *Trichostrongylus* (55%), and *Nematodirus* (51%). In general, mixed infections were observed, with 38% and 18% of flocks infected with three or all four genera, respectively.

**Conclusions:**

The results of this study indicate that GINs are widespread in Norway. *Teladorsagia circumcincta* seems to be present in most flocks based on this screening. Moreover, the results show that *Nematodirus* spp. infect lambs throughout the country, predominantly *N. battus,* and indicate that this nematode has become more abundant, which could lead to an increase in nematodirosis.

**Supplementary Information:**

The online version contains supplementary material available at 10.1186/s13028-024-00743-z.

## Background

Gastrointestinal nematodes (GINs) are important parasites in sheep worldwide. As these infections can cause disease and affect sheep welfare, they can lead to significant production losses [1]. All ovine GINs have a direct lifecycle and sheep are exposed to these parasites on pasture. The occurrence and distribution of infected hosts depend on climatic factors, such as temperature and humidity, as these influence the development and survival of the free-living stages of the parasite [2–4]. Nevertheless, the overall extent and severity of GIN infections depend on a complex interplay between parasites and host-related factors. Studies have been conducted worldwide to investigate the prevalence of various GINs and to address the associated challenges in sheep farming [5–7].

In Norway, the prevalence of GINs in small ruminants was last investigated between 2007 and 2010 and was based on identification of third stage larvae (L3) present in coprocultures from lambs [8]. The most common genera found in sheep were *Trichostrongylus/Teladorsagia* (80–98%), *Haemonchus* (6–31%) and *Nematodirus* (14–34%) in the three regions studied (northern, inland, and coastal). Identification by microscopy of L3 is both imprecise and time-consuming and requires skilled experienced personnel. Thus, in recent years, molecular methods have been increasingly used for the detection and quantification of parasite genera/species [9]. For example, a DNA amplification method, droplet digital polymerase chain reaction (ddPCR), has been established and used for precise detection and quantification of the three most important GINs in sheep in Sweden, i.e., *Haemonchus, Teladorsagia,* and *Trichostrongylus* [10–14]. By this method, the sample (extracted DNA) is partitioned into thousands of nano-sized oil droplets (compartments). Following PCR amplification, the droplets are evaluated by a droplet reader and detected as either positive or negative of the target molecule. Thereafter, the analysis program (Quantasoft) uses Poisson statistics to determine the concentration (copies/µL) of the target molecule. The method can be used to measure two targets of interest in the same reaction, by running a duplex assay. This enables the simultaneous quantitative analysis of both one species/genus-specific target, and one target specific for all strongyles, in the same reaction. Thus, the proportion of target species/genus of the total strongyles/nematodes can be measured in the sample. Other examples of valuable PCR-based tools for sensitive detection of GIN are the allele specific PCR, barcoding, and metabarcoding [15–18]. However, ddPCR offers certain advantages, such as no standard curves are required, little impact of PCR-inhibitors, as well as absolute quantification [10, 11, 19–21].

The aim of this study was to investigate the current occurrence of the major nematodes in lambs in selected areas of Norway, as determined by ddPCR. A second aim was to investigate the relative proportions of these nematodes at regional levels.

## Methods

### Selection of flocks

Flocks were recruited from the Norwegian sheep recording system, with data previously collected by a questionnaire as described earlier [22]. Briefly, the dataset comprised 1378 sheep farmers, and all counties of Norway were represented. The number of flocks in the dataset correlated well with the general distribution of the sheep population in Norway. All farmers were stratified by location (i.e., northern, central, eastern, southern, and western regions), and then a random selection of between 60 and 120 herds from northern, eastern, and western areas was made in Stata SE/16.0 (Stata Statistics/Data Analysis: Release 16. College Station, TX: StataCorp LLC). A prerequisite for selection was that farmers confirmed their participation in the project. In total, 257 farmers from the dataset were contacted for participation. In addition, some farmers were enrolled through meetings/project communication, including participants in the central and southern regions.

In total, samples from lambs were collected from 134 flocks. The sampled flocks were located in the northern (n = 24), eastern (n = 31), and western (n = 71) regions. In addition, some flocks from the central region (n = 7), and one flock from southern region were included (n = 1). The majority of flocks were randomly selected from the questionnaire dataset (66%), while about a third (34%) were enrolled as a result of meetings/project communication. The lambs had usually been treated with anthelmintics during the summer, and June was the most common time of deworming (48%). Nevertheless, a few had never been treated (10%), and most of these flocks were located in the northern region (data not shown).

### Sample collection

Sample collection took place in 2021 and 2022. Farmers were instructed to collect individual samples from 10 lambs in autumn (August/September), after the grazing period. Samples were taken directly from the rectum, either by hand or with a “faecal spoon” that was provided, together with an instruction leaflet, by the Norwegian University of Life Sciences (NMBU). The sampled animals had not been treated with anthelmintics during the 6 weeks prior to sampling. Samples were collected into airtight zip-lock bags and sent overnight by post to the laboratory. On arrival, the samples were numbered from 1 to 10 and processed individually. Samples that could not be processed the same day, were stored for no more than 2 days in a refrigerator at 4 ℃.

### Copromicroscopy

**Fig. 1:**
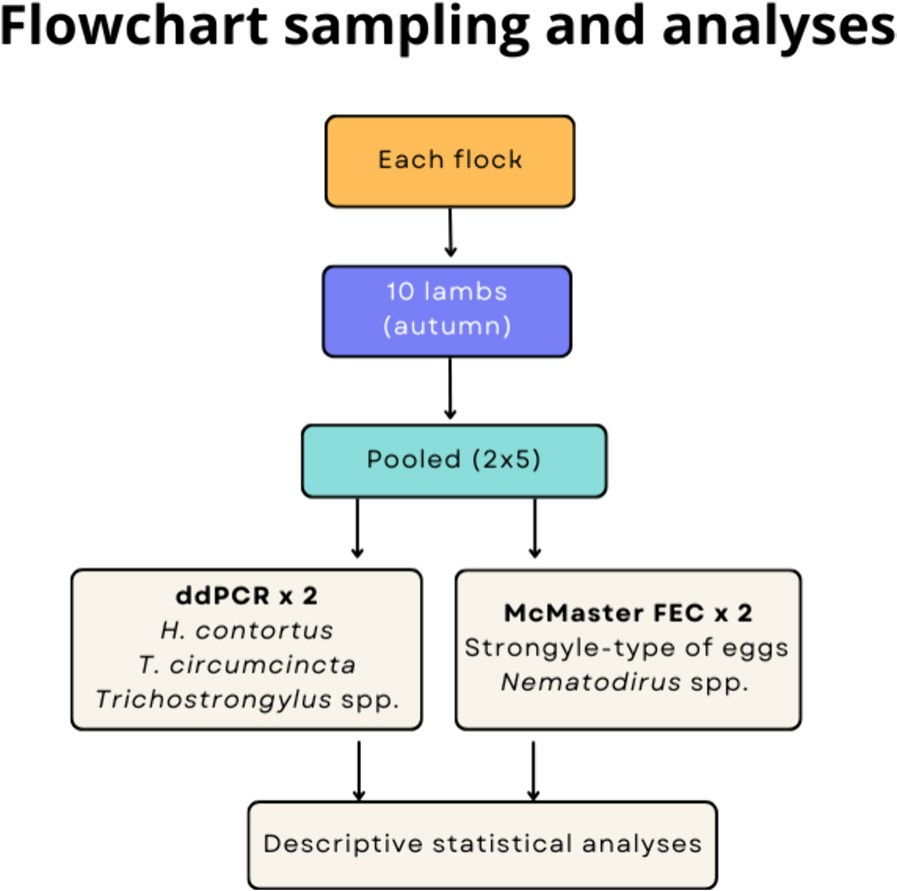
Overview of the sampling procedure and analysis on flock level. Two duplicate pooled samples were prepared, each duplex analysed with McMaster and ddPCR respectively

A modified McMaster method, with a theoretical detection limit of 50 EPG [23] was used to estimate the worm burden by the number of strongyle-type eggs per gram of faeces (EPG), and for detection of *Nematodirus* spp. eggs. Three grams from five individual samples / flock were pooled, to obtain two composite samples from each flock (Fig. 1). The five individual samples were selected randomly for the two pools. A total of 210 mL of water was then added to each of the pooled samples. After thorough mixing, the material was sieved through a metal strainer (150 µm mesh size) and collected in a bowl. The material obtained was collected in a 10 mL tube and centrifuged at 580 g for 3 min. After discarding the supernatant, saturated NaCl was added to the remaining faecal pellet and mixed by vortex. Using a plastic pipette, the suspension was transferred to a McMaster chamber and the eggs were counted at 40 × magnification.

### Coproculture

A total of two pooled coprocultures were prepared from each flock, using the same groups of individual samples as for copromicroscopy. Approximately 25 g of faeces (5 g from each of five animals in each pooled sample) were placed in plastic beakers. After thorough mixing with a wooden spatula, water was added to obtain a paste. The cultures were stored in an incubator at 25 ℃ for 2 weeks. Every second day, the cultures were mixed, and water was added to obtain approximately the same viscosity as the faecal paste on the day that the culture was first prepared.

### Larvae collection

A Baermann funnel was set up and filled with water. The faecal culture was wrapped in gauze and placed on top of the funnel. After 24 h, the larval suspension was collected in tubes (10 mL) and centrifuged at 580 g for 3 min. The supernatant was then aspirated, leaving approximately 1 mL of larval suspension in the tube. The precipitated L3 larvae were then stored at -18 ℃ prior to molecular analysis.

### Molecular analysis

#### DNA extraction

After thawing the frozen larvae at room temperature, DNA was extracted using the Nucleospin DNA stool kit (Macherey–Nagel, PA, USA) in combination with bead beating (FastPrep-24 5G, MP Biomedicals CA, USA). The method was applied according to the manufacturer’s protocol, using 220 µL of the larval suspension. The DNA templates obtained were frozen at −20 °C until molecular analysis. DNA from positive controls was obtained by the same extraction procedure, using morphologically identified adult male worms of: *Trichostrongylus colubriformis, Teladorsagia circumcincta,* and *Haemonchus contortus*; see Supplementary file 1.

#### Digital droplet PCR (ddPCR)

Duplex ddPCR assays were performed to detect *H. contortus*, *T. circumcincta* and *Trichostrongylus* spp., and to determine their relative proportion in mixed strongyle populations. As data on the occurrence of *Haemonchus* in these flocks has previously been described [24], they are only included for comparative purposes, whilst the relative proportion of *Haemonchus* is presented and discussed in full. The method was performed according to the previously described protocol [10], using 2 µL of DNA template (previously obtained/isolated from the 220 µL of larval suspension) from each of the larval cultures. Three reactions were prepared for each sample, each containing different primers and probe combinations specifically targeting a unique species-specific (i.e., *H. contortus, T. circumcincta*) or genus-specific (*Trichostrongylus*) region in the internal transcribed spacer region 2 (ITS-2) of the ribosomal RNA gene array, and another primers and probe set targeting a universal region in the ITS-2 that is common to all strongyles (Table 1). RNase-free water was used as a non-template control in each assay, along with a positive control of the specific genus being tested for. Baseline was established based on the negative and positive droplets of the controls. Samples that showed oversaturation were diluted 20-fold in RNase-free water. A threshold of 2.5 copies/µL was set to classify each sample as positive/negative. The threshold was determined in agreement with the limit of detection (LoD) of previous studies [10, 25], and test-assays using positive and negative controls, and dilutions thereof.

**Table 1:** Overview of primer/probe sequences used in ddPCR analyses

	Universal strongylid	*H. contortus*	*T. circumcincta*	*Trichostrongylus* spp.
F	GATTCGCGTATCGATGAAAAA	TGAACATGTTGCCACTATTTGAG	GAATGATATGAACGCGTATTGC	TGTTCCTGTATGATGTGAACGTG
R	CCGAAGGGAAAACCCAAC	TTAGTTTCTTTTCCTCCGCT	TTAGTTTCTTTTCCTCCGCT	TTAGTTTCTTTTCCTCCGCT
P	TGCAGACGCTTAGAGTGGTG	AATGCAACCTGAGCTCAGGC	GAGCTCAGGCGTGATTACCC	CGTGATTACCCGCTGAACTT
FP	HEX	FAM	FAM	FAM

Primers and probes used in duplex ddPCR assays, targeting the ITS-2 region [10]: universal with *H. contortus*/*T. circumcincta*/*Trichostrongylus* spp. *F* forward primer, *R* reverse primer, *P* probe, *Fp* flurophore

### Statistical analysis

After ddPCR, all results were analysed for the presence of positive droplets using Poisson statistics with the Quantasoft software (BioRad), which determined the concentration (copies/µL) and fractional abundance of the target molecule in each sample. The relative proportions of *H. contortus*, *T. circumcincta,* and *Trichostrongylus* were determined for all pooled samples to estimate their relative contributions to the nematode burdens in lambs. Firstly, the mean abundance of the two pooled samples were calculated for each of the respective target (i.e., *H. contortus*/Universal, *T. circumcincta*/Universal, *Trichostrongylus*/Universal). These were summed up to get the total proportion (TP) of the three nematodes combined. A multiplication factor (i.e., 100/TP) was used to calculate the mean relative proportion:

$${\text{relative }}\,{\text{proportion}}\,{\text{of}}\,{\text{target}}\,{\text{parasite}}\, = \,\frac{100}{{TP}}\, * \,{\text{mean}}\,{\text{proportion}}\,{\text{of}}\,{\text{target}}\,{\text{parasite}}$$

As for occurrence results of both ddPCR and copromicroscopic analysis, flocks were classified as positive for the respective GIN if one and/or both pooled samples were positive. Statistical analysis was then carried out using Excel (Microsoft), Stata (StataCorp), and R (Version 2023.12.0 + 369). Descriptive analysis was performed for each variable to generate means with standard deviations for continuous variables (EPG), and frequencies for categorical variables (pos/neg for the respective GIN). For calculations of significance based on frequencies (obtained from categorical variables), contingency table analysis (Pearson chi square test) was used. Logistic regression models were fitted to investigate the relationship of relevant explanatory variables with the presence of GINs as a binary outcome. For each GIN (i.e., *H. contortus*, *T. circumcincta*, *Trichostrongylus* spp., and *Nematodirus* spp.), the following explanatory variables were included: 1) year of sampling (i.e., 2021, 2022), 2) type of selection of flocks (random from questionnaire dataset, by meetings), and 3) region (i.e., northern, central, eastern, southern, and western). Explanatory variables that were not significant (P-value < 0.05) were eliminated, resulting in a final model for *H. contortus* and *Nematodirus* spp. with region as predictor variable; see Supplementary file 2. For all statistical analyses, a P-value < 0.05 was regarded as significant.

Maps were created using the symbol map by Datawrapper (www.datawrapper.de). The flowchart was designed using canva (www.canva.com).

## Results

### GIN occurrence and distribution

The overall GIN burden (i.e., mean number of excreted trichostrongyle eggs/EPG ± SD) was 1150 ± 1388 EPG, and gradually increased from north to south, with the highest values observed in samples from lambs located in western region (Table 2).

**Table 2:** Occurrence of gastrointestinal nematodes in Norwegian lambs

Area (no of flocks)	H. contortus*	95% CI	*Nematodirus* spp.**	95% CI	*T. circumcincta*	95% CI	*Trichostrongylus* spp.	95% CI	Mean EPG*** [range]	Mean EPG*** SD
Northern (n = 24)	2 (8)	1–27	16 (67)	45–84	24 (100)	86–100	12 (50)	29–71	546 [0–1575]	408
Central (n = 7)	2 (29)	4–71	5 (71)	29–96	7 (100)	59–100	3 (43)	10–82	796 [150–2400]	752
Eastern (n = 31)	22 (71)	52–86	20 (65)	45–81	28 (90)	74–98	19 (61)	42–78	1154 [100–4375]	1023
Western (n = 71)	53 (75)	63–84	28 (39)	28–52	66 (93)	84–98	39 (55)	43–67	1387 [0–9300]	1700
Southern (n = 1)	1 (100)		0		1 (100)		1 (100)			
Total (n = 134)	80 (60)	51–68	69 (51)	43–60	126 (94)	89–97	74 (55)	46–64	1150 [0–9300]	1388

^*^Previous results [24] included for comparison

^**^99% of *Nematodirus* ssp. were identified to be *N. battus* by microscopy

^***^Southern region is not included, due to only one flock sampled

Number of flocks (%) where H. contortus*, *Nematodirus* spp.**, *T. circumcincta*, and *Trichostrongylus spp.* were detected in pooled samples of lambs, divided by region. The mean EPG of strongyle-type of eggs, at regional level, are presented in the last two columns***

**Fig. 2:**
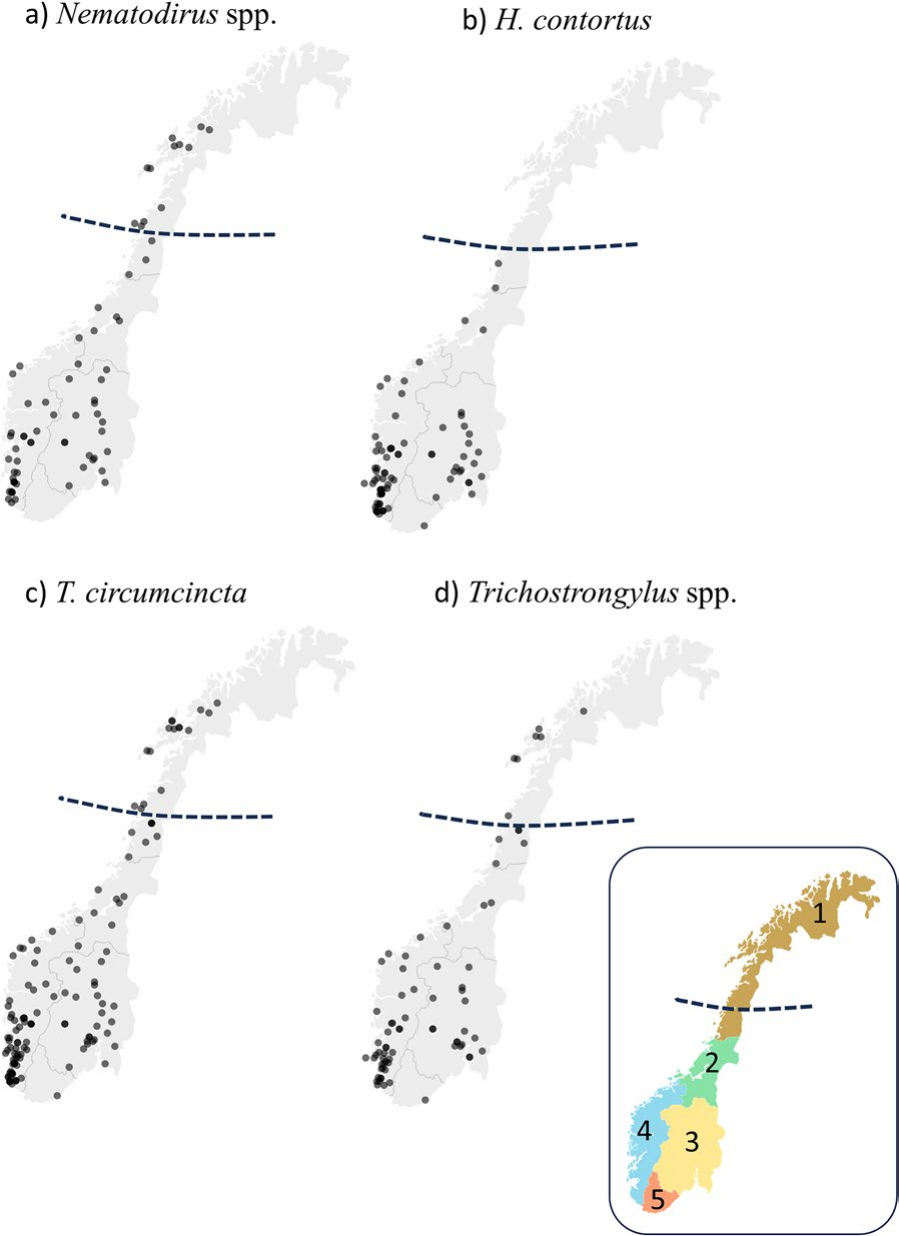
Geographical overview of gastrointestinal nematodes (GINs) in Norwegian lambs. Geographical overview of GINs: **a** *Nematodirus* spp. (n = 69), **b** *H. contortus* (n = 80), **c** *T. circumcincta* (n = 126) and **d** *Trichostrongylus* spp. (n = 74) detected in sampled lambs from a total of 134 flocks. The Arctic circle (66° 34’N) is indicated by the stapled lines. Regions of Norway are shown on colored map; northern (1), central (2), eastern (3), western (4), southern (5)

Eggs of *Nematodirus* spp. were found in about half of the samples from lambs (51%; Table 2). The most frequently observed species was *N. battus* (approximately 99%). This parasite showed a higher occurrence among sampled lambs from northern, central, and eastern regions (65–71%) than in the western region (39%; P < 0.05) (Table 2). The northernmost finding of *Nematodirus* spp. was in the northern region (in Troms and Finnmark county), with a latitude of 69.23°N (Fig. 2a).

The overall occurrence of *T. circumcincta* was 94%, and it was found in all sampled lambs from the northern and central region (100%; Table 2). There was a widespread geographic distribution of *T. circumcincta* (Fig. 2c), with no evident regional difference in occurrence (P > 0.05). A lower occurrence of *Trichostrongylus* spp. (55%) was found, but the geographical distribution was widespread, with the occurrence ranging from 43 to 61% in the various regions (Table 2, Fig. 2d). Furthermore, statistically significant variations in regional occurrence were not found (P > 0.05). In total, *H. contortus* was found to occur in 60% of the flocks, showing a highly significant variation in regional occurrence (P < 0.0001). The highest occurrence was found in western (75%) and eastern (71%) regions (Table 2, Fig. 2b; P < 0.0001). Of the GINs screened for, *T. circumcincta* and *Trichostrongylus* spp. were found furthest north at a latitude of 69.38°N (in Troms and Finnmark county). The year of sampling, or type of recruitment, did not show significant effects on the presence of any of the GINs (P > 0.05).

### Composition, relative proportion

Monospecific infection was found in only a few flocks (13%), of which *T. circumcincta* (82%) and *H. contortus* (18%) were the only species involved. Considering all four species/genera, most flocks (87%) had mixed multi-species infections (≥ 2 species/genera detected). Of these, the combination of two species/genera infections was detected in about a third of the flocks (31%), three species/genera infections were most frequently found (38%), and, less frequently, all four (18%). Of those flocks with mixed infections of three species/genera, the most common composition was with *H. contortus*, *T. circumcincta* and *Trichostrongylus* (49%), while a mix of *Nematodirus* and *T. circumcincta* was most frequently seen in flocks with two species/genera detected (44%).

**Fig. 3:**
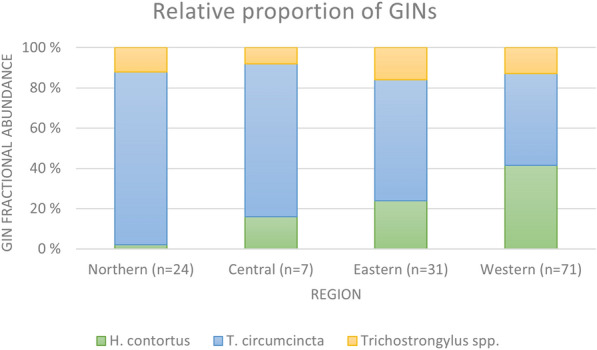
Relative proportion of gastrointestinal nematodes (GINs) in Norwegian lambs. Mean fractional abundance of GINs in samples from lambs, divided by region (n = 133*). * Southern region is not included due to only one flock sampled

The mean relative proportion of *H. contortus,* and *T. circumcincta,* showed regional differences (Fig. 3). The proportion of samples positive for *H. contortus* seems to gradually decrease from the western regions (41%), to eastern (24%), central (16%), and finally northern (2%) regions. In contrast, the proportion of *T. circumcincta* gradually increased from the western (46%), eastern (60%), central (76%), and northern (86%) regions. In general, *Trichostrongylus* spp. comprised the lower part of the nematode burden in all regions (8–16%).

## Discussion

This study shows that *T. circumcincta* was the most common nematode relative to the other nematodes investigated (i.e., *H. contortus*, *Trichostrongylus* spp., and *N. battus*) in Norwegian lambs at the end of the grazing period. This aligns with other studies in regions with a subarctic, humid climate, such as occurs commonly in northern Europe [7, 26, 27] and southeastern Canada [28]. In the previous Norwegian study, this nematode was also reported to be the most commonly detected GIN in lambs as detected by necropsy (75%) [8]. Although our results indicate an even higher occurrence than previously reported [8], this could be influenced by the sensitivity of method used (molecular analyses as opposed to microscopic identification). Nevertheless, it may also be related to factors influencing parasitic development (e.g., climate, animal density). Moreover, anthelmintic resistance (AR) in *T. circumcincta* has previously been detected in Norway [29, 30], which could have an impact on the prevalence.

Our results could indicate an increase in worm burden, as the overall mean number of trichostrongyle eggs shed was considerably higher (1150 ± 1388 EPG) than found in the previous nationwide survey (392 ± 714 EPG) [8]. Although the same modified McMaster technique was used in the previous study, it was done on individual samples and not on pooled samples as used in our investigation. Regardless of these variations in study design (i.e.,individual vs. flock level), the LoD was reportedly the same. Furthermore, previous studies have shown a high correlation between individual and pooled samples analysed by McMaster [31]. The possible increase in worm burden might be related to climatic changes, as both mean annual air temperature and precipitation in Norway have increased during the last decades [32]. However, this remains speculative as weather fluctuations from year to year could also influence the results. Another contributing factor could be related to an increased sheep density. Although the total number of sheep in Norway has remained stable over the last decade, the number of sheep holdings/farmers has decreased [33]. In other words, the mean flock size has increased during the last decade. This may have influenced sheep density during grazing, which, in turn, could increase the infection pressure of pasture parasites. Another potential factor is development of AR, which was first reported in 2012 [29, 30]. Nevertheless, it should be underlined that these studies are cross sectional. Thus, without data from a longitudinal study, trends regarding worm burden in lambs during the last decade are unknown. Furthermore, it is likely that a higher prevalence of *H. contortus* has an impact on the egg output, due to its prolific nature [34]. If so, our results suggest an increase in egg output/shedding, rather than a higher worm burden.

In the sampled flocks, *Trichostrongylus* spp. occurred less frequently in lambs than *T. circumcincta*. This contrasts with findings from other European countries, such as Germany [35], where *Trichostrongylus* was the predominant nematode. Factors, such as management, treatment regime, and season, can affect the composition and prevalence of GINs [36, 37]. For example, studies in Scotland [37] and Sweden [18] found that frequent whole-group treatment can reduce the species richness of the nematode population. This could be a possible explanation as to why only half of the lambs were infected with *Trichostrongylus*. However, data concerning drenching routines was limited in the present study. Anthelmintic resistance may also be a contributing factor, as it has previously been detected in *T. circumcincta* from sheep in Norway, but so far not in *Trichostrongylus* [29, 30]. Detection of *Trichostrongylus* was performed at the genus-level, and therefore it is likely that several species (i.e., *T. colubriformis*, *T. vitrinus*, *T. axei*) contributed to the category of *Trichostrongylus*, as all these species have been identified previously in Norwegian sheep flocks [8, 38]. In a previous study [8], the prevalence of *Trichostrongylus* spp. ranged from 3 to 22% (depending on species) in necropsied lambs. The prevalence was also estimated by using faecal material for coproculture, and then microscopic identification of L3. However, both *Trichostrongylus* spp. and *Teladorsagia* spp. were investigated as a combined/joint group by this method, which limits the comparison with our results.

*Nematodirus battus* was the most frequently observed *Nematodirus* species in our study (about 99%). This is an interesting finding as in Sweden this parasite is extremely rare and *N. fillicolis* predominates [39], indicating potential epidemiological differences regarding this parasite between these two quite similar, neighbouring countries. In Norway, *N. battus* was introduced in the late 1950s and has since spread to most regions [8, 40]. In a previous study [8], both necropsy examination and copromicroscopy were used to identify *N. battus* infection, of which worms were found mainly in necropsied lambs from the coastal region, while eggs were also detected in the northern region by faecal examination. Based on the McMaster method, the prevalence was found to be 14% (31/218) in the northern region [8]. The potential for comparison is somewhat limited since the previous study investigated the prevalence at the individual host level rather than at the flock level. Nevertheless, in our survey, the occurrence of *N. battus* was found to be 67% in the northern region. This could represent an increase in nematodirosis [41], thereby affecting lamb health and production. If the samples had been collected earlier in the year, the occurrence might have been even higher, as infection with this nematode occurs particularly in the spring under Norwegian conditions [8, 40]. However, this should be further investigated, given the large variations in egg hatching and seasonality of infection recently observed in the UK [42]. It seems clear that *N. battus* warrants more attention in Norway in the future, especially in the northernmost flocks.

Our results showed a high occurrence and relative abundance of *H. contortus* in sampled lambs from western and eastern regions. In the western parts, this could be related to a warmer, humid climate. It could also be related to sheep density, as both regions comprise the larger part of the total sheep population in Norway (> 70%). However, it may also be influenced by treatment practices, and potential anthelmintic resistance development. Resistance against anthelmintics has previously been detected in *H. contortus* in sampled sheep located in these regions [29, 30, 43], and a frequent occurrence of the resistance-associated allele (F200Y) has been reported from these regions [24]. Nevertheless, more research is required to elucidate this. The lower occurrence of *H. contortus* in the northern region is likely to be influenced by climate, as *H. contortus* is known to be cold sensitive [44]. Additional factors could also play a role. For instance, the treatment frequencies in flocks located in the northern region are lower than of those in other regions [45], thereby having a lower selection pressure and potential for AR to develop. Furthermore, AR has not been reported in *H. contortus* from the northern region [29, 43], and the resistance-associated allele (F200Y) has been found to occur rarely in this region [24]. These factors might contribute to the variation in prevalence. Nevertheless, it should be mentioned that the occurrence of other resistance-associated alleles in *H. contortus*, such as F167Y and E198A, have not been investigated in Norwegian sheep flocks. The prevalence of *H. contortus* in Norwegian lambs has previously been reported to be considerably lower (34%) [8] than found in our study (60%), which may indicate an increase in its spread. However, the different methods used (i.e., necropsy examination/total worm count versus molecular analysis) could have an impact on the sensitivity of detection. Furthermore, the nationwide prevalence of *H. contortus* during the last decade remains unknown.

Possible explanations for why *H. contortus* and *N. battus* vary between regions, but not those of *Trichostrongylus* spp. and *T. circumcincta*, could be due to the fact that the L3 of *Trichostrongylus* spp. and *T. circumcincta* are cold-resistant and have been reported to survive down to—10 °C [46, 47]. This would give them a general advantage in surviving under Nordic conditions. In contrast, the L3 of *H. contortus* are more sensitive to cold [44]. In contrast to other GINs, this parasite survives the winter within its host as an inhibited larva [48], whereas the eggs of *N. battus* rely on a chill exposure, before hatching at 10–12 °C [49]. These factors could play a role in the cross-sectional study of occurrence, as climatic conditions vary between regions. Other aspects to consider are lambing and grazing systems. In the north, lambing takes place later (about 1 month) than in the south. Furthermore, the turnout on spring pasture may also occur later due to snow on the pasture or limited pasture availability due to snow. Considering these points, the peak of L3 exposure on pasture could potentially occur later in the north, so the immunity of lambs could be less pronounced at the time of sampling than in lambs in southern areas.

Mixed multispecies infections occurred in most flocks. This aligns with other studies in Europe, where similar findings have been described [27, 35]. Although a multispecies infection was the norm at flock level, there is uncertainty whether this also applies on individual level, due to the pooling of samples.

The mean fractional abundance of GINs showed clear regional variations. *Teladorsagia circumcincta* dominated in northern region, which is probably related to the climate as this nematode is known to survive and develop at cold temperatures [47]. *Haemonchus contortus*, on the other hand, is cold sensitive [47], but survival within the host [7], combined with a humid climate in western region, can explain the higher mean fractional abundance of this area. In addition, studies have reported some degree of overwintering of *H. contortus* on pasture in Sweden and Scotland [48, 50]. Although the mean fractional abundances of *T. circumcincta* were lower in western and eastern regions, this might be related to *H. contortus* being a prolific egg-shedder, and thus, DNA from *T. circumcincta* larvae could appear low relative to DNA from *H. contortus* larvae in these regions. Additionally, this may also be related to other factors such as variation in treatment regimens, maintenance of refugia, grazing management, and pasture practices. Furthermore, the storage of some samples at 4 ℃ may have affected the species composition and abundance. As *Haemonchus* eggs are more susceptible to this temperature than *Teladorsagia* and *Trichostrongylus*, the quantity of infective larvae of *Haemonchus* in the pooled cultures may have been reduced [44]. In contrast, the prolific egg-production hallmark of *Haemonchus* could be reflected in a higher abundance of the larvae in coprocultures compared with the actual worm burden of the lambs [51], and with respect to comparison with less prolific GINs.

### Limitations

In this study, selection bias could have occurred as only those farmers that agreed to participate in the project were included in the selection pool. One could argue that those who have interest in the GIN infection status in their livestock may also have challenges related to GINs, and thereby do not represent a realistic picture of the current situation overall. Moreover, not all participants were selected randomly, as a third of them were included in the study due to project meetings, again potentially an indication of additional interest in GIN status in their livestock. Only a limited number of flocks from the central and southern regions were included, as they were not originally part of the scope of the study. Therefore, the occurrence of different GINs in these areas should be evaluated further.

In addition, farmers were specifically instructed to select individual lambs randomly. However, it cannot be guaranteed that some might not have been selected based on convenience or other factors (e.g., individuals with diarrhoea/reduced growth rate). Although the sample collection was not controlled, this was assessed to be the most feasible way to obtain material from a high number of flocks, with a widespread geographic location.

Some limitations related to the coprocultures were present: (1) approximately 25 g of faeces were required, however, some individual samples contained less than 5 g, (2) various amounts of water added during the incubation period, due to variations in the faecal consistency. These limitations were difficult to avoid, based on our study design and time feasibility. Therefore, the approach is considered to be semi-quantitative, where the results are an estimate of the relative abundance of the analysed species/genus. Metabarcoding could have been used as an alternative method for this purpose. Although this method doesn’t provide the absolute quantification of target-DNA, metabarcoding enables the total species mix within hosts to be determined [52]. However, this technique was not available at the time of our study.

## Conclusions

The study provides an update on the regional occurrence of the most important GINs in Norwegian lambs. Our results showed that, of the GINs screened for, *T. circumcincta* had the highest occurrence in sampled flocks. Moreover, the results indicate that *Nematodirus* spp. infect lambs throughout the country, predominantly *N. battus,* and suggest that this nematode has become more abundant, especially in the northern region. Furthermore, mixed multispecies infection appears to be common at flock level in all regions. Molecular methods are an emerging field within diagnostic parasitology. Our study suggests that ddPCR is a valuable tool to obtain more detailed results regarding the parasitic fauna in Norwegian sheep.

### Supplementary Information

Supplementary material 1.

Supplementary material 2.

## Data Availability

The raw data analysed in this study cannot be shared due to European General Data Protection Regulation. The cleaned/extracted datasets (with dummy variables) used and/or analysed during the current study are available from the corresponding author on reasonable request.
