# Supplementary material for: Occurrence of gastrointestinal nematodes in lambs in Norway, as assessed by copromicroscopy and droplet digital polymerase chain reaction
Source: Acta Vet Scand. 2024 May 25;66:22. doi: 10.1186/s13028-024-00743-z (PMC11127287; doi:10.1186/s13028-024-00743-z)
Supplement: Supplementary file 1 — Supplementary material 1. [file 13028_2024_743_MOESM1_ESM.pdf]

Supplementary file

Positive controls used in ddPCR assays

**Genus/species**	**Origin**	**Stage**	**Infection**
Teladorsagia circumcincta	Mouflon caroux (France)	Adult	Natural
Trichostrongylus colubriformis	Goat (France)	Adult	Monospecific
Fasciola hepatica	Norwegian White sheep (Norway)	Adult	Natural
Haemonchus contortus	Sheep (Slovakia)	Adult	Monospecific
