# Supplementary material for: Occurrence of gastrointestinal nematodes in lambs in Norway, as assessed by copromicroscopy and droplet digital polymerase chain reaction
Source: Acta Vet Scand. 2024 May 25;66:22. doi: 10.1186/s13028-024-00743-z (PMC11127287; doi:10.1186/s13028-024-00743-z)
Supplement: Supplementary file 2 — Supplementary material 2. [file 13028_2024_743_MOESM2_ESM.pdf]

Supplementary file 2

The logistic regression formula can be written as: $ln\ (\pi\ /\ (1 - \pi))\ = \beta 0 + \beta 1 x 1 + \beta 2 x 2 + \cdots \beta k x k$

where π is the probability of category A occurs

Response variable: "Hc", dichotomous outcome indicating the presence of *Haemonchus contortus* (1 = positive, 0 = negative)

Explanatory variable: "REGION", factor with 5 levels

1 = northern

2 = central

3 = eastern

4 = western

5 = southern

Commands used in R studio:

```
> glm.obj.3 = glm(Hc~factor(REGION), data=R_studio_data, family=binomial)
> summary(glm.obj.3)
```

Output:

```
Coefficients:
                 Estimate Std. Error z value Pr(>|z|)
(Intercept)       -2.3979     0.7385  -3.247  0.00117 **
factor(REGION)2    1.4816     1.1160   1.328  0.18431
factor(REGION)3    3.4095     0.8461   4.030 5.59e-05 ***
factor(REGION)4    3.4965     0.7871   4.442 8.90e-06 ***
factor(REGION)5   17.9640  1455.3977   0.012  0.99015
```

Response variable: "Nem", dichotomous outcome indicating the presence of *Nematodirus* spp. (1 = positive, 0 = negative)

Explanatory variable: "REGION", factor with 5 levels

1 = northern

2 = central

3 = eastern

4 = western

5 = southern

Commands used in R studio:

```
> glm.obj.4 = glm(Nem~factor(REGION), data=R_studio_data, family=binomial)
> summary(glm.obj.4)
```

Output:

```
Coefficients:
                  Estimate Std. Error z value Pr(>|z|)
(Intercept)      6.931e-01  4.330e-01   1.601   0.1094
factor(REGION)2  2.231e-01  9.421e-01   0.237   0.8128
factor(REGION)3 -1.177e-15  5.809e-01   0.000   1.0000
factor(REGION)4 -1.204e+00  4.967e-01  -2.424   0.0154 *
factor(REGION)5 -1.526e+01  8.827e+02  -0.017   0.9862
```
